# International veterinary epilepsy task force consensus report on epilepsy definition, classification and terminology in companion animals

**DOI:** 10.1186/s12917-015-0461-2

**Published:** 2015-08-28

**Authors:** Mette Berendt, Robyn G. Farquhar, Paul J. J. Mandigers, Akos Pakozdy, Sofie F. M. Bhatti, Luisa De Risio, Andrea Fischer, Sam Long, Kaspar Matiasek, Karen Muñana, Edward E. Patterson, Jacques Penderis, Simon Platt, Michael Podell, Heidrun Potschka, Martí Batlle Pumarola, Clare Rusbridge, Veronika M. Stein, Andrea Tipold, Holger A. Volk

**Affiliations:** Department of Veterinary Clinical and Animal Sciences, Faculty of Health and Medical Sciences, University of Copenhagen, Frederiksberg C, Denmark; Fernside Veterinary Centre, 205 Shenley Road, Borehamwood, SG9 0TH Hertfordshire, UK; Department of Clinical Sciences of Companion Animals, Utrecht University, Yalelaan 108, 3583 CM Utrecht, The Netherlands; Clinical Unit of Internal Medicine Small Animals, University of Veterinary Medicine, Veterinärplatz 1, 1210 Vienna, Austria; Department of Small Animal Medicine and Clinical Biology, Faculty of Veterinary Medicine, Ghent University, Salisburylaan 133, Merelbeke, 9820 Belgium; Animal Health Trust, Lanwades Park, Kentford, Newmarket, CB8 7UU Suffolk, UK; Centre for Clinical Veterinary Medicine, Ludwig-Maximilians-University, Veterinärstr. 13, 80539 Munich, Germany; University of Melbourne, 250 Princes Highway, Weibee, 3015 Victoria, Australia; Section of Clinical & Comparative Neuropathology, Centre for Clinical Veterinary Medicine, Ludwig-Maximilians-University, Veterinärstr. 13, 80539 Munich, Germany; Department of Clinical Sciences, College of Veterinary Medicine, North Carolina State University, 1052 William Moore Drive, Raleigh, NC 27607 USA; University of Minnesota College of Veterinary Medicine, D426 Veterinary Medical Center, 1352 Boyd Avenue, St. Paul, MN, 55108 USA; Vet Extra Neurology, Broadleys Veterinary Hospital, Craig Leith Road, Stirling, FK7 7LE Stirlingshire, UK; College of Veterinary Medicine, University of Georgia, 501 DW Brooks Drive, Athens, GA 30602 USA; Chicago Veterinary Neurology and Neurosurgery, 3123 N. Clybourn Avenue, Chicago, IL 60618 USA; Department of Pharmacology, Toxicology and Pharmacy, Ludwig-Maximillians-University, Königinstr. 16, 80539 Munich, Germany; Department of Animal Medicine and Surgery, Veterinary Faculty, Universitat Autònoma de Barcelona, Campus UAB, Bellaterra, 08193 Barcelona, Spain; Fitzpatrick Referrals, Halfway Lane, Eashing, Godalming, GU7 2QQ Surrey, UK; School of Veterinary Medicine, Faculty of Health & Medical Sciences, University of Surrey, Guildford, GU2 7TE Surrey, UK; Department of Small Animal Medicine and Surgery, University of Veterinary Medicine Hannover, Bünteweg 9, 30559 Hannover, Germany; Department of Clinical Science and Services, Royal Veterinary College, Hatfield, AL9 7TA Hertfordshire, UK

**Keywords:** Epilepsy, Seizures, Dog, Classification, Semiology

## Abstract

Dogs with epilepsy are among the commonest neurological patients in veterinary practice and therefore have historically attracted much attention with regard to definitions, clinical approach and management. A number of classification proposals for canine epilepsy have been published during the years reflecting always in parts the current proposals coming from the human epilepsy organisation the International League Against Epilepsy (ILAE). It has however not been possible to gain agreed consensus, “a common language”, for the classification and terminology used between veterinary and human neurologists and neuroscientists, practitioners, neuropharmacologists and neuropathologists. This has led to an unfortunate situation where different veterinary publications and textbook chapters on epilepsy merely reflect individual author preferences with respect to terminology, which can be confusing to the readers and influence the definition and diagnosis of epilepsy in first line practice and research studies.

In this document the International Veterinary Epilepsy Task Force (IVETF) discusses current understanding of canine epilepsy and presents our 2015 proposal for terminology and classification of epilepsy and epileptic seizures. We propose a classification system which reflects new thoughts from the human ILAE but also roots in former well accepted terminology. We think that this classification system can be used by all stakeholders.

## Background

Epilepsy is a complex brain disease where sudden and abnormal activity in neuronal networks causes the prominent clinical sign of seizures characterised by motor, autonomic and/or behavioural features. Epileptic seizures are episodic and brief (in most cases less than 2–3 min). Epilepsy can rise from a plethora of causes. A few rare cases are purely genetic (e.g. channelopathies), some are developmental and have complex genetic and epigenetic influences (e.g. neuronal migration disorders) and some are caused by injury to the brain (e.g. trauma, infectious, inflammatory, vascular or neoplastic disease). In a significant number of cases, the cause is not clear. Although the mechanisms behind companion animal epilepsy are largely uncovered, it is clear that epilepsy in some purebred dogs is a direct result of a genetic defect, where seizures are the core clinical sign of disease. This has been described for the Lagotto Romagnolo, Belgian shepherd and Boerboels [1–4]. A high epilepsy prevalence in a specific breed or the accumulation of epileptic individuals within specific dog families are strong indicators of inherited epilepsy, but often it is unknown if genetic defects are the sole cause of the epilepsy or if the epilepsy might arise from multifactorial causal influences including environmental, developmental, provoking and genetic factors, and similar issues apply to human cases [5].

The true prevalence of epilepsy in dogs is unknown and has been estimated to be 0.6–0.75 % in the general dog population [6, 7]. Epidemiological population prevalence studies in specific breeds with idiopathic epilepsy has been conducted in the Labrador retriever (3.1 %), Belgian shepherd (9.4 %) and petit Basset Griffon vendeen (8.9 %), and pedigree studies in the Boxer, Irish wolfhound, English Springer spaniel, Vizsla, Bernese mountain dog, Standard poodle, Belgian shepherd, Border collie, Australian shepherd and Border terrier among others, and these have provided evidence for inherited epilepsy [8–25] (for further information on epilepsy related to specific breeds see Hülsmeyer et al. [26]). Given the heterogeneity of epilepsy where causes and signs of disease are greatly variable, a standardised terminology and classification system for epilepsy is crucial in order to provide accurate descriptive information for diagnostic and communicative processes.

Since 1964 epilepsy and epileptic seizures have in human medicine been organized and categorized in a classification and terminology system, published by the International League Against Epilepsy—ILAE [27–33]. The ILAE sees itself as *“the world's preeminent association of physicians and other health professionals working towards a world where no persons’ life is limited by epilepsy”* and the noble mission of this organization is *“to ensure that health professionals, patients and their care providers, governments, and the public world-wide have the educational and research resources that are essential in understanding, diagnosing and treating persons with epilepsy”* (ILAE homepage www.ilae.org).

The 1985 classification of seizure types and the 1989 classification of the epilepsies continues to be used worldwide in human epilepsy [32, 34]. However, in recent years, the ILAE classification task force has taken to continually updating and revising on the subject of epilepsy producing new consensus documents approximately every 5 years with the most recent being published in 2010 and a further version is currently under much debate [27]. The terminology and classification framework they provide is seen as an ever evolving process, reflecting the constant gain in our understanding of the disease and the complications inherent in ambiguous, misinterpreted and potentially stigmatising vocabulary. This has generated considerable discussion amongst their members reflecting the complexity of the task.

In veterinary medicine, a number of classification proposals for canine epilepsy have been published over the years always reflecting in part the current ILAE proposals (e.g. [35–38]). There remains, however, a lack of a consensus, a common ‘language’, for the classification and terminology used between veterinary and human neurologists and neuroscientists, practitioners, neuropharmacologists and neuropathologists. Companion animal epilepsy papers reflect a variety of modifications of the definitions of epilepsy and epileptic seizures derived from the core classification documents of the ILAE (e.g. [1, 7, 9–15, 17, 23–25, 27, 28, 30–36, 38–46]. This has led to an unfortunate situation where different veterinary publications and textbook chapters on epilepsy merely reflect individual author preferences with respect to terminology, which can be confusing for the reader. Put in a scientific and educational perspective, the existing lack of uniformity with respect to definitions and terminology furthermore represents a major problem, as comparisons between research studies are compromised. Even more important, it prevents the implementation of a common understanding of epilepsy and standardized professional guidelines which can help clinicians when diagnosing animals with epilepsy and advising the owners.

Terminology and classification of epilepsy should be a ‘user friendly’, reliable and valid tool for the benefit of different users and the patient [47]. There is a ‘chain of care’ from the pet’s owner through the primary clinician to neurology specialists and researchers. The language should be concise to reduce errors and to simplify conversation. The first opinion veterinarian and specialists alike should be able to use the classification framework to manage the disease appropriately and to communicate with others using the same language. In the age of the internet, the pet owner also needs to be able to, at least in part, comprehend the terminology used. The scientist should find in the classification a pragmatic and reliable instrument to investigate the disease’s aetiology, pathophysiology, treatment, and outcome. Finally terminology should reflect current knowledge and not be changed for the sake of change, which has recently also been emphasized by Shorvon (2014) [5] and others with respect to the ILAE discussions on classification. Otherwise important historical data might be lost and in some cases it might be wise to stay with terms which are in widespread use and have stood a test of time instead of a rapid change of terminology emanating from academic discussions [5]. Considering these issues, terminology and classification needs to fulfil dissimilar and sometimes antagonistic needs and at the same time be adaptable to change.

In adopting the terms associated with human epilepsy we should acknowledge some of the very significant differences when applying it to small animal patients. We cannot interview our patients as in human medicine and the clinician’s interpretation of the patient’s seizure signs are invariably restricted to the owner's description and often a poor quality video(s). Typically electroencephalography (EEG) is an impractical tool in animals and so cannot contribute to a general classification scheme—in contrast to the situation in human medicine. Furthermore some owners will decline diagnostic investigation due to financial concerns or because some procedures require general anaesthesia, and therefore the owners’ report of seizure history and phenomenology, supported by digital (video) recordings, remain the most central diagnostic epilepsy marker in companion animals (in fact as it does in humans).

Another discrepancy between people and animals which must be addressed is the assessment of possible impairment of consciousness during seizures. The determination of consciousness impairment is challenging and often very important to people because of personal and public safety. The ILAE classification cites consciousness as an important factor for diagnosis. Veterinary medicine is better aligned with the challenges facing paediatric medicine in this regard.

This consensus statement group aims to provide the veterinary community with a proposal focused on the classification of epilepsy and epileptic seizures. To ensure we have all stakeholders involved, the consensus-working group is composed of veterinary and human neurologists and neuroscientists, practitioners, neuropharmacologists and neuropathologists.

### Proposal

In recent years the ILAE has been able to advance human epilepsy classification to a sophisticated level as a consequence of advanced diagnostics and the uncovering of an increasing number of mechanisms (including genetic) that lead to epilepsy. The definition of epilepsy accepted by the ILAE in 2005 reflected the immense progress with respect to the identification of aetiologies and the understanding of seizure generation. Here an epileptic seizure was defined as: *“A transient occurrence of signs and/or symptoms due to abnormal excessive or synchronous neuronal activity in the brain”,* and epilepsy was defined as: *“A disorder of the brain characterized by an enduring predisposition to generate epileptic seizures and by the neurobiologic, cognitive, psychological, and social consequences of this condition. The definition of epilepsy requires the occurrence of at least one epileptic seizure”* [30]*.* In 2010 the ILAE refined the definitions even further [27]; to demonstrate the on-going discussions in the ILAE, it has recently been proposed that epilepsy should be considered a true disease of the brain [48]. This contradicts the previous understanding of epilepsy as a condition of the brain—or a collection of signs from the brain. Berg and Scheffer stated back in 2011 [28]: “These proposals are not meant to be permanent, but form part of a transition to a system that will ultimately allow for meaningful translation of scientific understanding to the classification of the epilepsies for clinical and other purposes”. Although we need to move veterinary epilepsy classification forward we also must, as in human medicine, consider carefully whether any change of terminology is meaningful [5]; we must accept that currently we have not advanced the scientific understanding of the mechanisms leading to epilepsy in companion animals to the level applicable to the ILAE.

In the following sections the IVETF discusses the current understanding of companion animal epilepsy and presents our 2015 proposal for terminology and classification of epilepsy and epileptic seizures. We propose a classification system which reflects new thoughts from the human ILAE but also has its roots in former well accepted terminology. We think that this classification system can be used by all stakeholders. The classification has two elements: (a) an aetiological element and (b) a seizure type classification.

## Definitions

In human medicine, epilepsy can often be confirmed by electroencephalography (EEG) although epileptic people may have a normal EEG and an abnormal EEG can also exist in people without epilepsy. The use of EEG in veterinary medicine is currently of questionable routine clinical value and therefore our definitions should be seen as clinically operational and reflecting expert knowledge in the field with respect to the typical appearance of epileptic seizures and their symptomatology. This is not a major drawback, and for definition purposes, it is reasonable (in human and animal epilepsy) to define seizures and epilepsy by the clinical appearance of seizures. Please also refer to the glossary for further definitions not listed in the main text.

### Seizure

The term can be used for any sudden, short lasting and transient event. It does not imply that the event is epileptic.

### Epileptic seizure

Manifestation(s) of excessive synchronous, usually self-limiting epileptic activity of neurons in the brain. This results in a transient occurrence of signs which may be characterized by short episodes with convulsions or focal motor, autonomic or behavioural features and due to abnormal excessive and/or synchronous epileptic neuronal activity in the brain.

### Reactive seizure

A reactive seizure is a seizure occurring as a natural response from the normal brain to a transient disturbance in function (metabolic or toxic in nature)—which is reversible when the cause or disturbance is rectified. A provoked seizure can be considered as being synonymous with a reactive seizure.

### Epilepsy

Epilepsy is defined as a disease of the brain characterized by an enduring predisposition to generate epileptic seizures. This definition is usually practically applied as having at least two unprovoked epileptic seizures >24 h apart [48].

## Classification

Historically veterinary medicine has operated with various terminology/synonyms for epilepsy types defined by aetiology and phenotypic manifestations which are themselves defined by the distribution of abnormal electrical activity in the brain. The development of ILAE classifications and changes in veterinary epilepsy terminology throughout time are detailed in Table 1 and 2.

**Table 1:** Veterinary terminology and its most common amendments over time

	Early terminology	Terminology currently in use	Suggested veterinary terminology 2015
**EPILEPTIC SEIZURES**			
**An epileptic seizure with clinical signs indicating activity which starts in a localised area in the brain**	Petit Mal	Partial/focal seizure	Focal epileptic seizure^a^
	Aura	- Simple partial/focal seizure (consciousness unimpaired^a^)	
-Will present with focal motor, autonomic or behavioural signs alone or in combination			
		- Complex partial/focal seizure (consciousness impaired^a^)	
**An epileptic seizure with clinical signs indicating activity involving both cerebral hemispheres from the start.** -In dogs and cats the seizure presents predominantly as immediate ‘convulsions’ and loss of consciousness. Salivation, urination and/or defecation often also occur during convulsions. May also (but rare) present as atonic or myoclonic seizures	Grand Mal (always implicating convulsions)	Primary generalized seizure	Generalized epileptic seizure
**An epileptic seizure which starts in a localized area in the brain and spreads subsequently to involve both hemispheres.** -In dogs and cats the seizure starts with localized motor, autonomic and/or behavioural signs rapidly followed by convulsions. Salivation, urination and/or defecation often also occur during convulsions.	Partial seizure with secondary generalization (secondary generalized seizure)	Focal seizure with secondary generalization	Focal epileptic seizure evolving to become generalized
**EPILEPSY**			
Epilepsy classified by aetiology	Primary Epilepsy	Idiopathic Epilepsy	Idiopathic Epilepsy
	- Epilepsy where no structural cerebral pathology is suspected	- Epilepsy where no structural cerebral pathology is suspected. A genetic component may be involved	1. Proven genetic background
			2. Suspected genetic background
			3. Unknown cause and no indication of structural epilepsy
Epilepsy classified by aetiology	Secondary or Acquired epilepsy	Symptomatic Epilepsy	Structural epilepsy
	- Epilepsy caused by identified cerebral pathology	- Epilepsy caused by identified cerebral pathology	- Epilepsy caused by identified cerebral pathology
Epilepsy classified by aetiology	Cryptogenic	Probably or possibly symptomatic epilepsy	Unknown cause
	- Meaning hidden	- A suspected symptomatic cause, which however remains obscure	

^a^Regarding our ability to evaluate if consciousness is unimpaired or impaired during focal seizures (former termed simple and complex focal seizures). We recommend that it is not attempted to interpret signs occurring during focal seizures where dogs may appear e.g. confused, unable to recognize the owner or not responding to commands as impaired consciousness, as this cannot objectively be investigated in animals

**Table 2:** Development of ILAE classifications

ILAE 1981 and 1989	ILAE 2010	Berg and Scheffer 2011 [28]
Generalized seizures are those in which the first clinical and electroencephalographic changes indicate initial involvement of both cerebral hemispheres.	Generalized seizures are conceptualized as originating at some point within and rapidly engaging bilaterally distributed networks. Such bilateral networks can include cortical and subcortical structures, but do not necessarily include the entire cortex. Generalized seizures can be asymmetric.	
Focal seizures (previously defined as partial) are those in which the first clinical and electroencephalographic changes indicate initial activation of a system of neurons limited to a part of one cerebral hemisphere.	Focal seizures are conceptualized as originating at some point within networks limited to one hemisphere. They may be discretely localized or more widely distributed. Focal seizures may originate in subcortical structures. For each seizure type, ictal onset is consistent from one seizure to another, with preferential propagation patterns that can involve the contralateral hemisphere. The Glossary of Ictal Semiology [51] Provides an initial vocabulary which, while in need of revision and expansion, is an example of the type of “dictionary” needed for discussing seizure semiology. This not only allows but requires greater precision in seizure description. Terms such as hypermotor, akinetic, versive, hemiconvulsion, and retained responsiveness or awareness communicate much more information about a patient’s seizure manifestations than do the terms complex and simple partial.	
Idiopathic epilepsy: there is no underlying cause other than a possible hereditary predisposition.	Genetic epilepsy: the epilepsy is, as best as understood, the direct result of a known or presumed genetic defect(s) in which seizures are the core symptom of the disorder. This attribution must be supported by specific forms of evidence (e.g. specific molecular genetic studies or family studies).	Genetic: The epilepsy is a direct result of a genetic cause. Ideally, a gene and the mechanisms should be identified; however, this term would also apply to electroclinical syndromes for which twin or family segregation studies reproducibly show clinical evidence of a genetic basis (e.g., in the case of the genetic generalized epilepsies). At this time, channelopathies are the best example of genetic epilepsies. Ultimately, we expect causes of epilepsies to be identified by the mechanisms involved (i.e., channelopathies, mitochondrial respiratory chaindefects, etc.).
Symptomatic epilepsy: this type of epilepsy is the consequence of a known or suspected disorder of the central nervous system.	Structural and metabolic epilepsy: this type of epilepsy is the secondary result of a distinct structural or metabolic condition. These structural or metabolic disorders may be of acquired or genetic origin (as is the case for malformations of cortical development and certain metabolic disorders).	Structural-Metabolic: The epilepsy is the secondary result of a separate structural or metabolic condition. Structural and metabolic were combined to separate the concept from genetic and also because the two are often inseparable. Note that structural brain lesions, including many malformations of cortical development, often have genetic causes and most metabolic disorders are also of genetic origin. The distinction between “genetic epilepsy” and epilepsy due to a structural/metabolic cause is far from perfect, but we anticipate more specific characterizations of cause in the upcoming years.
Cryptogenic (probable symptomatic) epilepsy: this refers to a disorder whose cause is hidden or occult. Cryptogenic epilepsies are presumed to be symptomatic.	Unknown epilepsy: the nature of the underlying cause is as yet unknown; it may have a fundamental genetic basis (e.g., a previously unrecognized channelopathy) or it may be the consequence of an unrecognized structural or metabolic disorder not yet identified.	Unknown: Plain and direct, this label simply and accurately indicates ignorance and that further investigation is needed to identify the cause of the epilepsy. Unlike cryptogenic (presumed symptomatic), it makes no presumptions and requires no explanation or reinterpretation.

### Epilepsy types defined by aetiology

#### Idiopathic epilepsy

##### Idiopathic epilepsy

(idiopathic defined as a disease in its own right, per se) should be seen as the overarching and bridging term, which can be sub-classified into three sub-groups reflecting the advancements in the field:

1. Idiopathic epilepsy (genetic epilepsy)—a causative gene for epilepsy has been identified/confirmed genetic background
2. Idiopathic epilepsy (suspected genetic epilepsy)—a genetic influence supported by a high breed prevalence (>2 %), genealogical analysis and/or familial accumulation of epileptic individuals^*,**^.
  ^*^Shorvon stated in 2014 [5]*: “It seems very likely that the genetic influences in idiopathic epilepsies probably are complex involving multiple genes and interactions between genes (epistatic) and between genes and the environment (epigenetic)”.*
  ^**^A list of breeds with a high epilepsy incidence or prevalence compared to the general background population can be found in Hülsmeyer et al. [26]*. Please note that the epilepsy status within breeds may fluctuate over time and furthermore be influenced by differences between countries (e.g. due to preferences with respect to currently popular breeding lines).*
3. Idiopathic epilepsy (epilepsy of unknown cause)—epilepsy in which the nature of the underlying cause is as yet unknown and with no indication of structural epilepsy.

Please see consensus on Diagnostic approach to epilepsy in dogs [49] for further information on diagnostic work-up.

#### Structural epilepsy

##### Structural epilepsy

is characterized by epileptic seizures which are provoked by intracranial/cerebral pathology including vascular, inflammatory/infectious, traumatic, anomalous/developmental, neoplastic and degenerative diseases confirmed by diagnostic imaging, cerebrospinal fluid examination, DNA testing or post mortem findings (see consensus on Diagnostic approach to epilepsy in dogs [49]). Lafora disease progressive myoclonic epilepsy would be classified under structural epilepsy as the gene defect results in a storage disease which alters the brain structurally and where the epileptic seizures associated with the structural changes in the brain are one of the multiple clinical and neurological signs associated with the primary storage disease [50].

### Classification by seizure semiology (seizure type classification)

#### Focal epileptic seizures

Focal epileptic seizures are characterized by lateralized and/or regional signs (motor, autonomic or behavioural signs, alone or in combination). The ictal onset is consistent from one epileptic seizure to another. They may be discretely localised or more widely distributed. Focal epileptic seizures may originate in subcortical structures, with preferential propagation patterns that can involve the contralateral hemisphere. With focal epileptic seizures, the abnormal electrical activity arises in a localized group of neurons or network within one hemisphere. The clinical signs reflect the functions of the area or areas involved.

Focal epileptic seizures can present as:

- Motor (episodic focal motor phenomena e.g. facial twitches, repeated jerking head movements, rhythmic blinking, twitching of facial musculature or repeated rhythmic jerks of one extremity)
- Autonomic (with parasympathetic and epigastric components e.g. dilated pupils, hypersalivation or vomiting)
- Behavioural (focal epileptic seizure activity which in humans can represent psychic and/or sensory seizure phenomena may in animals result in a short lasting episodic change in behaviour such as e.g. anxiousness, restlessnesss, unexplainable fear reactions or abnormal attention seeking/‘clinging’ to the owner.

#### Generalized epileptic seizures

Generalized epileptic seizures are characteried by bilateral involvement (both sides of body and therefore both cerebral hemispheres involved). Generalized epileptic seizures may occur alone or evolve from a focal epileptic seizure start. In dogs and cats generalized epileptic seizures predominantly present as tonic, clonic or tonic-clonic epileptic seizures. As a rule the animal will lose consciousness during convulsive epileptic seizures (myoclonic seizures excluded). Salivation, urination and/or defecation furthermore often also occur (myoclonic seizures excluded).

Generalized convulsive epileptic seizures involving bilateral motor activity

- Tonic-clonic
- Tonic
- Clonic
- Myoclonic (Jerking movements usually affecting both sides of the body)

Non-convulsive generalized epileptic seizures

- Atonic (also called ‘drop attacks’—sudden and general loss of muscle tone which usually cause the animal to collapse)

#### Focal epileptic seizures evolving into generalized epileptic seizures

Focal epileptic seizures can spread from initial regional cerebral involvement to bilateral cerebral involvement. The seizure will start with regional motor, autonomic and/or behavioural signs and then rapidly be followed by a convulsive stage with bilateral tonic, clonic or tonic-clonic activity and loss of consciousness. This is the most common seizure type observed in the dog. The onset of the focal epileptic seizure is often very short (seconds to minutes) after which follows the secondary generalisation with convulsions. The focal epileptic seizure onset may be difficult to detect due to its brief nature. When taking the seizure history, clients should be interviewed thoroughly about what (or if something) happens before convulsions (please see De Risio et al. [49] for further information on diagnostic work-up).

### The semiological description of an epileptic seizure

If classification is to be carried out according to seizure type, it makes sense to have a systematic way of describing seizures. The evolution of the seizure over time is what is important.

#### Phases associated with epileptic seizures

The epileptic seizure is classified as the ictus (seizure activity)—followed by a postictal phase (where the normal brain function is restored). The ictus may consist of a generalized epileptic seizure alone, a focal epileptic seizure alone, or a focal epileptic seizure which evolves into a generalized seizure. In the postictal phase, the brain restores its normal function. The postictal phase may be very short or last for several hours to days. Typically, the animal is disoriented, may have behavioural abnormalities such as repetitive vocalisation, compulsive locomotion failing to avoid obstacles, be tired, ataxic, hungry or thirsty, express a need to urinate, defecate or appear exhausted and sleep for a longer period of time. Postictal blindness or aggression may also be present.

#### Prodrome

In some animals (but not so common), ictus may be preceded by a so-called prodrome, a long-term (hours to days) change in disposition and indicator of forthcoming seizures. Humans may experience days of, for example, irritability, withdrawal or other emotional aberrations. In dogs the most common prodromal signs described are hours or days of restlessness, anxiousness, appear as being irritated (e.g. with uncharacteristic aggression towards other pets), or attention-seeking behaviour which is known to the owner as a long-term marker of a forthcoming seizure episode. Prodromes (if present) can represent a potential important therapeutic window for pulse therapy. Prodromal signs must be discriminated from focal seizure signs. Prodromal signs are defined by their long lasting nature whereas focal seizures which may display similar signs when they occur alone or before generalized convulsive seizures are very short (seconds to minutes).

#### Consciousness in focal epileptic seizures

Variable to no impairment of consciousness may appear during focal seizures. However, we propose that no attempt should be made to evaluate if consciousness is unimpaired or impaired (previously described as simple or complex focal (or partial) seizures respectively). Although animals may appear as if consciousness is impaired during focal epileptic seizures (awake but being confused, not recognizing the owner, not responding to commands) we cannot assess this objectively. It will always be a subjective interpretation in animals that cannot report what they are experiencing. Therefore it is not meaningful to subclassify focal epileptic seizures using consciousness.

### Agreed modified glossary of descriptive terminology for ictal semiology in accordance with the ILAE guidelines (based on Blume et al. [51])

The glossary of descriptive terminology was discussed within the IVETF in 2014. The terms that the majority of the group (>50 % of 95%CI from 14 raters) felt could be also used to describe ictal semiology are listed below:

#### I. General terms

1.0 **Semiology** That branch of linguistics concerned with clinical signs.
2.0 **Epileptic seizure** Manifestation(s) of excessive synchronous, usually self-limiting epileptic activity of neurons in the brain. This results in a transient occurrence of signs which may be characterized by short episodes with convulsions or focal motor, autonomic or behavioural features and due to abnormal excessive or synchronous epileptic neuronal activity in the brain.
3.0 **ICTUS** A sudden neurological occurrence such as a stroke or an epileptic seizure.
4.0 **Epilepsy** Epilepsy is defined as a disease of the brain characterized by an enduring predisposition to generate epileptic seizures. This definition is usually practically applied as having at least two unprovoked epileptic seizures >24 h apart [48].
5.0 **Focal epileptic seizures** are conceptualized as originating within networks limited to one hemisphere. They may be discretely localized or more widely distributed. Focal epileptic seizures may originate in subcortical structures. For each epileptic seizure type, ictal onset is consistent from one seizure to another, with preferential propagation patterns that can involve the contralateral hemisphere [27].
6.0 **Generalised epileptic seizure** An epileptic seizure whose initial semiology indicates, or is consistent with, more than minimal involvement of both cerebral hemispheres. Generalized epileptic seizures are conceptualized as originating at some point within, and rapidly engaging, bilaterally distributed networks [27].
7.0 **Convulsion** Primarily a lay term. Episodes of excessive, abnormal muscle contractions, usually bilateral, which may be sustained or interrupted.

#### II. Terms describing epileptic seizure semiology

These are descriptors of seizures unless specified otherwise.

1.0 **Motor** Involves skeletal musculature resulting in any phenotypic manifestation. The motor event could consist of an increase (positive) or decrease (negative) in muscle contraction to produce a movement. Unless noted, the following terms are adjectives modifying “motor seizure” or “seizure” e.g. “tonic motor seizure or dystonic seizure”, and whose definitions can usually be understood as prefaced by: “refers to …”.
  1.1.1 **Tonic** A sustained increase in muscle contraction lasting a few seconds to minutes.
    1.1.1.2.1 **Versive** A sustained, forced conjugate ocular, cephalic and/or truncal rotation or lateral deviation from the midline.
    1.1.1.2.2 **Dystonic** Sustained contractions of both agonist and antagonist muscles producing athetoid or twisting movements which when prolonged may produce abnormal postures.
  1.1.2 **Myoclonic (adjective); Myoclonus (noun)** Sudden, brief (<100 msec) involuntary single or multiple contraction(s) of muscles(s) or muscle groups of variable topography (axial, proximal limb, distal).
    1.1.2.1 **Clonic** Myoclonus which is regularly repetitive, involves the same muscle groups, at a frequency of about 2–3 seconds, and is prolonged. Synonym: rhythmic myoclonus.
  1.1.3 **Tonic-clonic** A sequence consisting of a tonic followed by a clonic phase. Variants such as clonic-tonic-clonic may be seen.
    1.1.3.1 **Generalised tonic-clonic epileptic seizure** (Formerly "Grand Mal" Seizure) Noun: Bilateral symmetrical tonic contraction then bilateral clonic contractions of somatic muscles usually associated with autonomic phenomena.
  1.1.4 **Atonic** Sudden loss or diminution of muscle tone without an apparent preceding myoclonic or tonic event lasting one to two seconds or more, involving head, trunk, jaw or limb musculature.
  1.1.5 **Synchronous (Asynchronous)** Motor events occurring (not) at the same time or at the same rate in sets of body parts.

1.2 **Automatism** Noun: A more or less coordinated, repetitive, motor activity usually occurring when cognition is impaired and for which the subject is usually amnesic afterwards. This often resembles a voluntary movement, and may consist of inappropriate continuation of ongoing preictal motor activity.
  The following adjectives are usually employed to modify “automatism”.
  1.2.1 **Oroalimentary** Lip smacking, lip pursing, chewing, licking, teeth grinding or swallowing.
  1.2.2 **Pedal** Indicates principally distal component involvement, bilateral or unilateral. Usually running movement.

2.0 **Non-motor**

2.1 Aura* Noun: A ‘subjective’ ictal phenomenon that, in a given patient, may precede an observable seizure; if alone, constitutes a sensory seizure. This can result in behavioral changes such as fear, aggression, searching behaviour, attention, body sensation.

*What is an aura? Commonly owners report that they can foresee a motor seizure when specific, and to the owner, well known signs repeatedly appear within seconds or minutes prior to convulsions. The term aura has in the past been used to describe such a forewarning of convulsions. This term originated from human epileptology where aura in early ILAE classifications was used “to denote symptomatology that encompasses subjective sensory phenomena as well as vegetative signs (for example, the epigastric sensations accompanying mesial temporal epilepsy)” and thus did not include motor phenomena. The group recommends that the term aura is not used in veterinary medicine. The signs occurring as the first indication of seizure activity (marking the beginning of ictus) and interpreted by the dog owner as a warning sign is indeed a focal seizure onset and should be referred to as such.

2.2 **Autonomic** A sensation consistent with involvement of the autonomic nervous system, including cardiovascular, gastrointestinal, sudomotor, vasomotor and thermoregulatory functions. In companion animals, salivation, mydriasis, urination and/or defecation may commonly be observed.
3.0 **Somatotopic modifiers**

3.1 **Laterality**
  3.1.1 **Unilateral** Exclusive or virtually exclusive involvement of one side as a motor, sensory or autonomic phenomenon.
    3.1.1.1 **HEMI-** A prefix to other descriptors e.g. hemiclonic.
  3.1.2 **Generalised** (syn. “bilateral”) More than minimal involvement of each side as a motor or autonomic phenomenon.
    Motor component: further modified as:
    3.1.2.1 **Asymmetrical** Clear distinction in quantity and/or distribution of activity on the two sides.
    3.1.2.2 **Symmetrical** Virtual bilateral equality in these respects.

3.2 **Body part** Refers to area involved i.e. limb, face, trunk and other.
  3.2.1 **Axial** Involves trunk, including neck.
  3.2.2 **Proximal limb** Signifies involvement from shoulders to metacarpus, hip to metatarsus.
  3.2.3 **Distal limb** Indicates involvement of paws.

4.0 **Modifiers and descriptors of epileptic seizure timing** The following terms are listed in the form (adjective, noun, verb) according to principal usage; as adjective unless specified.
4.1 **Incidence** Noun: Refers to the number of epileptic seizures within a time period or the number of seizure days per unit of time.
  4.1.1 **Regular, irregular** Consistent (inconsistent) or predictable (unpredictable, chaotic) intervals between such events.
  4.1.2 **Cluster** Incidence of epileptic seizures within a given period (usually one or a few days) which exceeds the average of incidence over a longer period for the patient. Cluster seizures can be defined clinically as two or more seizures within a 24-h period.
  4.1.3 **Provocative factor** Noun: Transient and sporadic endogenous or exogenous element capable of augmenting seizure incidence in the patient with chronic epilepsy and evoking seizures in susceptible non-epileptic individuals.
    4.1.3.1 **Reactive** A reactive seizure is a seizure occurring as a natural response from the normal brain to a transient disturbance in function (metabolic or toxic in nature)—which is reversible when the cause or disturbance is rectified. A provoked seizure can be considered as being synonymous with a reactive seizure.
    4.1.3.2 **Reflex** Objectively and consistently demonstrated to be evoked by a specific afferent stimulus or by activity of the patient. Afferent stimuli can be: elementary, i.e. unstructured (light flashes, startle, a monotone sound) or elaborate i.e. structured, (a symphony). Activity may be elementary, e.g. motor (a movement).

5.0 **Duration** Time between the beginning of initial epileptic seizure manifestations, such as the e.g., focal epileptic seizure signs or full body convulsions to the cessation of experienced or observed epileptic seizure activity. Does not include non-specific seizure premonitions or postictal states.
5.1 **Status epilepticus** An epileptic seizure which shows no clinical signs of arresting after a duration encompassing the great majority of seizures of that type in most patients or recurrent epileptic seizures without resumption of baseline central nervous system function interictally. Status epilepticus can be defined clinically as (a) greater than 5 min of continuous epileptic seizures or (b) two or more discrete epileptic seizures between which there is incomplete recovery of consciousness (for generalized convulsive seizures).
6.0 **Severity** A multicomponent assessment of an epileptic seizure by observers and the patient.

Components primarily of observer assessment include: duration, extent of motor involvement, impairment of interaction with environment intra-ictally, maximum number of seizures per unit of time.

7.0 **Prodrome** A pre-ictal phenomenon. A subjective or objective clinical alteration that heralds the onset of an epileptic seizure but does not form part of it. Prodrome is a long lasting event (hours to days) and should not be confused with focal onset seizure signs which are very brief events (seconds to minutes).
8.0 **Postictal phenomenon** A transient clinical abnormality of central nervous system function that appears or becomes accentuated when clinical signs of the ictus have ended.
8.1 **Lateralising (TODD‘S (or bravais’)) phenomenon** Any unilateral postictal dysfunction relating to motor, somatosensory and/or integrative functions including visual, auditory or somatosensory.
8.1 **Non-lateralising phenomenon** Behavioural changes such as fear, aggression, increased appetite.

## Abbreviations

IVETF: International Veterinary Epilepsy Task Force
ILAE: International League Against Epilepsy
